# Effects of Exogenous Sodium Nitroprusside Spraying on Physiological Characteristics of Soybean Leaves at the Flowering Stage under Drought Stress

**DOI:** 10.3390/plants12081598

**Published:** 2023-04-10

**Authors:** Zhipeng Qu, Yumei Tian, Xinyu Zhou, Xiaomei Li, Qi Zhou, Xiyue Wang, Shoukun Dong

**Affiliations:** 1 Agricultural College, Northeast Agricultural University, Harbin 150030, China; 2 Agriculture and Food Science and Technology Branch, Heilongjiang Agricultural Engineering Vocational College, Harbin 150025, China

**Keywords:** soybean, nitric oxide, drought stress, drought resistance physiology

## Abstract

Nitric oxide (NO) plays a significant role in plant drought resistance. However, the effects of the exogenous application of NO to crops under drought stress vary within and among species. In this study, we explored the influence of exogenous sodium nitroprusside (SNP) on the drought resistance of soybean leaves in the full flowering stage using two varieties: drought-tolerant HN44 and non-drought-tolerant HN65. Spraying SNP on soybean leaves at the full flowering period under drought stress improved the NO content in soybean leaves. The activities of nitrite reductase (NiR) and nitrate reductase (NR) in leaves were affected by NO inhibition. The activity of antioxidant enzymes in leaves increased with the extension of SNP application time. Contents of osmomodulatory substances, including proline (Pro), soluble sugar (SS), and soluble protein (SP) increased gradually with the extension of SNP application time. The malondialdehyde (MDA) content decreased as the NO content increased, thus reducing membrane system damage. Overall, spraying SNP reduced damage and improved the ability of soybean to cope with drought. This study explored the physiological changes of SNP soybean under drought stress and provided theoretical basis for improving drought-resistant cultivation of soybean.

## 1. Introduction

Soybean (Glycine max (Linn.) Merr.) originated in China and is a highly valuable crop. In recent years, soybean production has been affected by drought. *The Impact of Disasters and Crisis on Agriculture and Food Security* [1] reported that drought is the chief cause of reductions in agricultural production. Under drought stress, levels of reactive oxygen species (ROS) increase, and plants respond to ROS-induced stress via enzymes in the antioxidant system, such as ascorbate oxidase (APX), peroxidase (POD), catalase (CAT), and superoxide dismutase (SOD) [2]. During drought, the extracellular osmotic potential of plants increases. Plants adjust the intracellular accumulation of proline (Pro), soluble protein (SP), soluble sugar (SS), and other substances to sustain the penetrant of cells; additionally, stomata close to reducing the excessive loss of moisture [3].

The application of exogenous growth regulator is an important strategy to improve the capacity of plants to deal with drought. For example, applying SNP to bananas under drought stress can effectively increase the ability of bananas to cope with drought [4]. Nitric oxide (NO) is a significant molecule in the plant response to drought stress. SNP is usually used as NO carrier in actual production to resist plant damage caused by adversity [5]. Adverse conditions will accelerate NO accumulation in cells, increasing the stress resistance of plants by enhancing the antioxidant system and osmoregulation [6]. For example, the application of sodium nitroprusside (SNP) to peach fruit under low−temperature stress reduces the activity of DNA methyltransferase (DNMT), thereby enhancing cold resistance [7].

The activity of antioxidant enzymes is also regulated by NO. Studies have shown that NO mediates the activity of antioxidant enzymes through S-nitrosylation, nitration or oxidation [8]. Spraying SNP on plants can improve antioxidant activity in plants to reduce the negative effects of ROS. For example, spraying SNP on soybeans under salt stress improves the activity of antioxidase in soybeans [9]. NO can also directly scavenge ROS, and plants can reduce plant damage through the coordination of the antioxidant enzyme system and NO [10]. Drought can cause membrane lipid peroxidation and increase membrane permeability [11]. The malondialdehyde (MDA), the product of membrane lipid peroxidation, content is affected by NO. In particular, Spraying SNP can decrease membrane lipid peroxidation and reduce the effects of drought stress on plants [12]. Spraying SNP can increase the accumulation of proline (Pro), soluble protein (SP), soluble sugar (SS), and other osmoregulatory substances in plants and reduce damage. It can increase levels of osmoregulatory substances in wheat treated with sodium chloride and alleviate the osmotic imbalance [13]. The process of nitrate degradation in cells is divided into two steps. The first step is the degradation of nitrate into nitrite by nitrate reductase. The second step is nitrite reductase to degrade nitrite into ammonium or nitric oxide, and nitrate reductase can also catalyze nitrite to produce nitric oxide [14,15], and NiR is the only nitrogen oxide reductase in rhizobia [16]. Nitrate reductase (NR) can catalyze nitrite to produce NO in soybean [17]. NR and NiR synergistically regulate NO in plants to increase plant stress resistance [18]. When the NO content in plants is low, the activities of NiR and NR will increase, especially under water-deficient conditions [15]. Alternatively, when the NO content is high, the activities of NiR and NR will be inhibited, thereby hindering the inorganic nitrogen metabolism pathway [19].

SNP is sensitive to different plants and even to different varieties in the same plant in practice. For the effective application of SNP, it is important to consider differences in sensitivity among species [14]. Furthermore, it is necessary to optimize the SNP concentration to improve plant stress resistance. In this experiment, using the drought type Heinong 44 (HN44) and sensitive type Heinong 65 (HN65) as experiment materials, the effects of SNP as the carrier of NO on the antioxidant ability and osmoregulation on soybean leaves at the full bloom stage were evaluated. The study results provide theoretical support for improved soybean cultivation in drylands.

## 2. Materials and Methods

### Plant Materials and Test Design

The previous research screened the drought tolerance of the main recommended soybean (Glycine max (Linn.) Merr.) varieties in Heilongjiang Province. The results showed that soybean cultivar HN44 was drought type and HN65 was a sensitive type, both from the Soybean Research Institute of Heilongjiang Academy of Agricultural Sciences [20]. The test was carried out at Northeast Agricultural University (E126.730, N45.745).

In this experiment, pots of 40 cm high and 10 cm in diameter were used. They were filled with river sand, and six soybean seeds of uniform size were planted in each pot. Before the opposite true leaves were fully expanded, 500 mL of water was provided every day. Opposite true leaves were fully expanded, leaving three seedlings with the same growth potential in each pot. Plants were treated with Hoagland nutrient solution [21] at 500 mL each time. During the full flowering stage (R2), the plants were subjected to drought stress with nutrient solution containing PEG-6000 (every day) and sprayed with SNP. (R2: Flowering on either of the two segments with fully grown leaves at the uppermost part of the main stem of soybean). Since the flowering period is a time when soybeans are sensitive to water demand due to their nutritional growth, flowering, and pod setting, the flowering period is chosen for the experiment, as described in Table 1.

Sampling was carried out at 8:00–9:00 in the morning on days 1, 3, 5, and 7 after the first SNP spray, and SNP solution was sprayed once 3 h before each sampling. Only one sample is taken for each treatment, and put into liquid nitrogen, and transferred to −80 °C. Samples were stored in the refrigerator until testing. For measurements, each treatment was repeated 3 times, and average values were obtained.

## 3. Test Methods

### 3.1. Determination of the NO Content

The NO content in soybean leaves was measured by the Greiss reagent method, Briefly, 0.1 g of fresh leaves and distilled water were added to a centrifuge tube. The tube was closed, put in water (100 °C, 30 min), and cool down to 25 °C, and added to a water bath at 100 °C again. Then, 0.5 mL of the supernatant and 0.5 mL of Greiss reagent were obtained [22].

### 3.2. Determination of Antioxidant Enzyme Activity

Superoxide dismutase (SOD) activity was assayed by the nitrogen blue tetrazolium method [23]. Peroxidase (POD) activity was assayed by the guaiacol method [24]. Ascorbate oxidase (APX) activity was assayed by ultraviolet spectrophotometry [25]. Briefly, 0.1 g of sample (except leaf veins) and 1 mL of extraction medium (supplemented with a small amount of CaCO_3_ for POD measurements) were added to a mortar at 4 °C, ground into a homogenate with a pestle, and then centrifuge the homogenate at 4 °C and 10,000× *g* for 15 min. The supernatant was collected and measured following the above method. The extraction medium for superoxide dismutase (SOD) was 50 mmol/L, phosphate buffer (pH 7.8), and 1% polyvinylpyrrolidone (PVP). The extraction medium for POD was distilled water. APX extraction medium was phosphate-buffered saline (50 mmol/L, pH 7.0) containing 0.1 mmol/LEDTA-Na_2_. Catalase (CAT) activity was measured following the methods described by Spychalla [26]. To prepare the reaction solution, 0.1 g sample was added to a centrifuge tube. A volume of PBS (0.15 M, pH 7.0, 200 mL) was added, followed by 0.3092 mL of 30% H_2_O_2_ and shaken vigorously.

### 3.3. Determination of Contents of Osmoregulatory Substances 

The proline (Pro) content was determined by the acid ninhydrin chromogenic method [27]. The soluble protein (SP) content was assayed by the Coomassie Brilliant Blue G-250 staining method [27]. The soluble sugar (SS) content was assayed by anthrone colorimetry [27]. Briefly, 0.1 g of sample (without leaf veins) and 1 mL of 80% ethanol were added to the mortar and ground into a homogenous slurry with a pestle. The mixture was filtered twice with activated carbon filter paper and transferred to a test tube. Then, 0.2 g of zeolite was added, followed by 5 min of strong shock. The supernatant was removed for centrifugation at 3000× *g* (10 min), and the supernatant was collected for the measurement of the proline (Pro) content by the above method. Additionally, 0.1 g of sample (without leaf veins) and 1 mL of distilled water were added to the mortar and ground into a homogenate with a pestle. Following centrifugation at 3000 r/min for 10 min, the supernatant was collected, and the above method was used to measure the contents of soluble protein and soluble sugar. The difference between soluble protein and soluble sugar is that the latter needs to be measured in water (20 min, 100 °C).

### 3.4. Determination of NiR and NR Activity

Nitrite reductase (NiR) activity was determined following previously described methods [28]. In a mortar with a temperature of 4 °C, 1 mL of a phosphate buffer solution (0.1 mol/L and pH 7.5) was added and ground into a homogenate with a pestle. The homogenate was centrifuged at 4 °C and 12,000× *g*. Measurements were obtained after 20 min.

Nitrate reductase (NR) activity was determined by in vivo method [29]. In a 50 mL beaker, 1.0 g of fresh leaves was added, a glass bottle stopper was pressed on the leaves, 1 mL of 0.3 g/mL trichloroacetic acid solution was added, and 9 mL of 0.1 mol/L potassium nitrate was added. After mixing, vacuum treatment and aeration treatment were performed several times for 1 min. After the leaves were completely softened and sunk to the bottom of the cup, they were sealed with nitrogen gas. Cups were maintained at room temperature for 30 min in the dark, and the above-mentioned trichloroacetic acid was added to the remaining treatments, and the color reaction.

### 3.5. Determination of MDA Content

The malondialdehyde (MDA) content was assayed following the methods of Wang [30]. Briefly, 0.1 g of sample and 1 mL of 10% trichloroacetic acid were added to a mortar for grinding, followed by centrifugation at 12,000× *g* for 10 min. Then, thiobarbituric acid (TBA) at 0.2 mL and 0.67% was added to the homogenate (0.4 mL), the samples were maintained in a water bath at 100 °C, mixed, and boiled for 30 min. After cooling, samples were centrifuged twice and the supernatant was obtained to evaluate absorbance at 450, 532, and 600 nm.

### 3.6. Data Analysis

Microsoft Office 365 (Home edition), IBM SPSS Statistics 27.0.1 (IBM Corporation, Armonk, NY, USA) and OriginPro 2021 (OriginLab Corp., Northampton, MA, USA) performed deviation calculation, significance analysis, and histogram drawing, respectively.

## 4. Results and Analysis

### 4.1. Changes in the NO Content in Soybean Leaves

**Figure 1:**
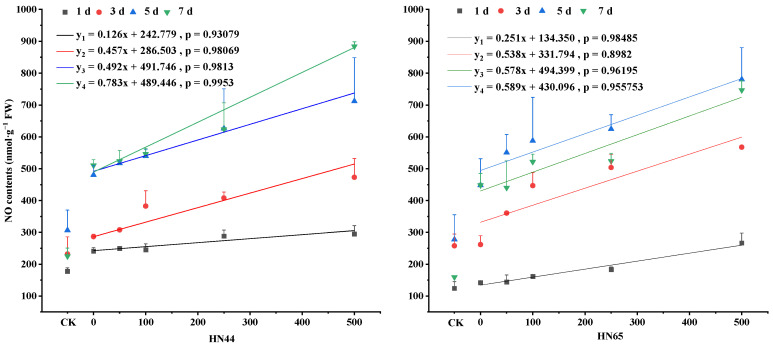
Changes in NO content during SNP mitigation of drought stress. NO stands for nitric oxide. The *X*-axis, CK represents the control, and the remaining numbers are SNP concentrations sprayed. Values (mean ± SE) are the averages of three independent experiments.

After drought treatment, the NO contents in soybean leaves increased. In HN44 the NO content in plants increased gradually as the SNP concentration increased. Over time, the NO content tended to increase. In HN44 at 5 days, the NO contents in soybean leaves treated with S50, S100, and S250 differed from those at 7 days by 1.27%, 1.23%, and −0.44%. The NO content tended to be stable. Compared with normally watered soybeans, the NO content in HN44 increased by 35.56%, 23.56%, 56.57%, 126.87% on days 1, 3, 5, and 7 under drought treatment (Figure 1).

In HN65 the NO content in plants increased gradually as the SNP concentration increased (The NO content of S1 treatment was lower than that of S0 at 7 day). Over time, the NO content tended to increase initially and then decrease, with a maximum value on day 5. Compared with normally watered soybeans, the NO content in HN65 increased by 14.40%, 1.46%, 61.49%, 180.85% at the 1st, 3rd, 5th, and 7th days under drought treatment. At 5 days, The NO contents in leaves treated with S50, S100, and S250 differed from those at 7 days by 22.88%, 31.16%, 39.29%, and 74.10%, respectively (Figure 1).

The NO content in HN44 was greater than that in HN65. Compared with that under normal watering, the NO content of the treatment group was higher in the drought−resistant variety HN44 on days 1 and 3 and was higher in the sensitive variety HN65 on days 5 and 7 (Figure 1).

### 4.2. Changes in NiR and NR Activity in Soybean Leaves

**Figure 2:**
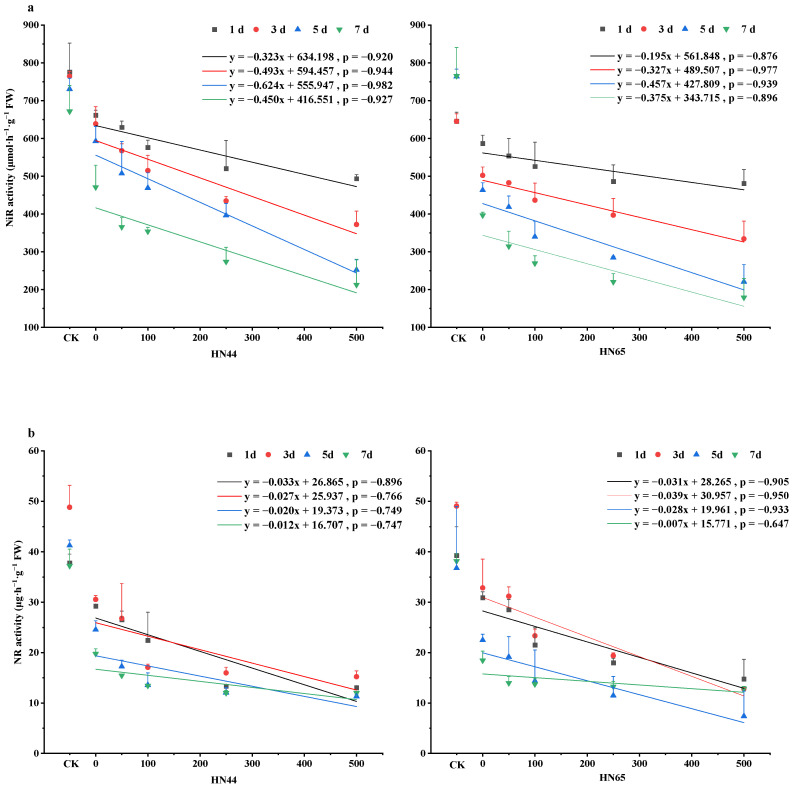
Changes in NiR and NR activities during NO mitigation of drought stress. (**a**) nitrite reductase (NiR) activity, (**b**) nitrate reductase (NR) activity. The *X*-axis, CK represents the control, and the remaining numbers are SNP concentrations sprayed. The Values (mean ± SE) are the averages of three independent experiments.

After soybeans were subjected to drought stress, the nitrite reductase (NiR) and nitrate reductase (NR) activities decreased. Compared with normally watered soybeans, NiR activity decreased by 14.75%, 16.44%, 18.87%, 29.89% in HN44 at 1, 3, 5, and 7 days, and decreased by 9.08%, 22.16%, 39.35%, 48.16% in HN65 under drought treatment. For NR, drought stress decreased the NR activity 22.70%, 37.40%, 40.78%, 46.87% in HN44 on days 1,3,5, and 7 and 21.40%, 33.00%, 38.84%, 51.62% in HN65, respectively. The activity of NiR decreased with the extension of time, while the activity of NR increased first and then decreased with the extension of time, and the maximum value appeared on the third day (Figure 2).

As shown in Figure 2a, the S500 treatment resulted in the greatest reduction in NiR activity. Compared with that for S0 in HN44, NiR activity in the S500 group decreased by 25.39%, 41.70%, 57.43%, and 54.78% at four time points. Similarly, enzyme activity in HN65 decreased by 18.06%, 33.42%, 52.32%, and 54.84%. NiR activity decreased gradually over time. NiR activity in HN44 was greater than that in HN65, and there were important differences in drought tolerance and the effects of spraying of different concentrations of SNP between the two varieties.

As shown in Figure 2b, the activity of NR was lowest on day 5 at 11.31 μmol·h^−1^·g^−1^. Compared with those in S0 on day 5, the activity of NR in the drought-tolerant variety HN44 decreased by 29.78%, 44.78%, 50.88%, 53.93% for S50, S100, S250, and S500, respectively. NR activity decreased gradually and finally stabilized as the SNP concentration increased. In the sensitive variety HN65, NR activity decreased by 14.85%, 36.11%, 48.91%, and 67.09% for S50, S100, S250, and S500. After drought treatment and spraying with different concentrations of SNP, there were significant differences between HN44 and HN65.

### 4.3. Changes in Antioxidant Enzyme Activity in Soybean Leaves

**Figure 3:**
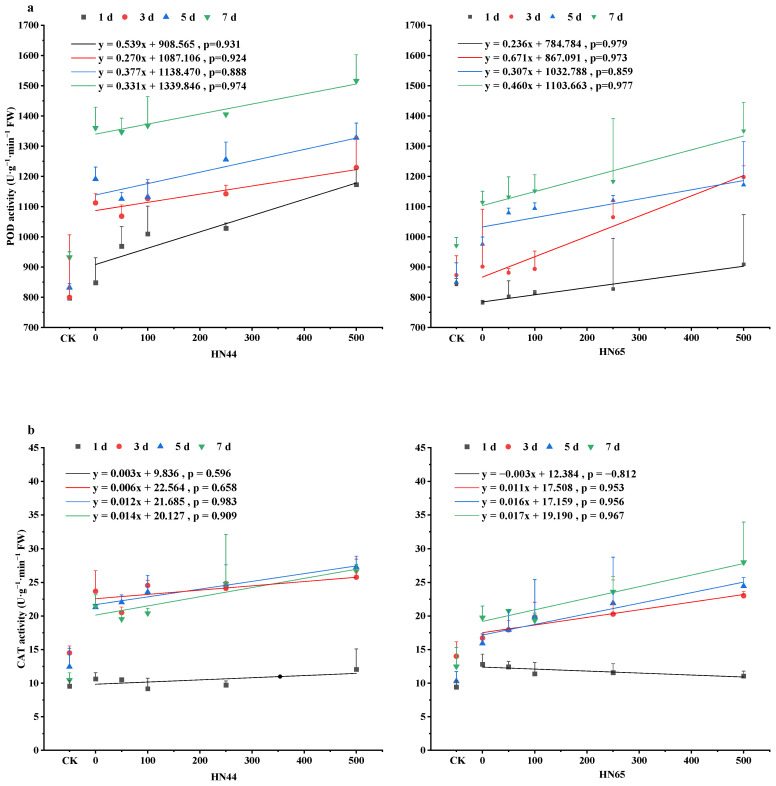

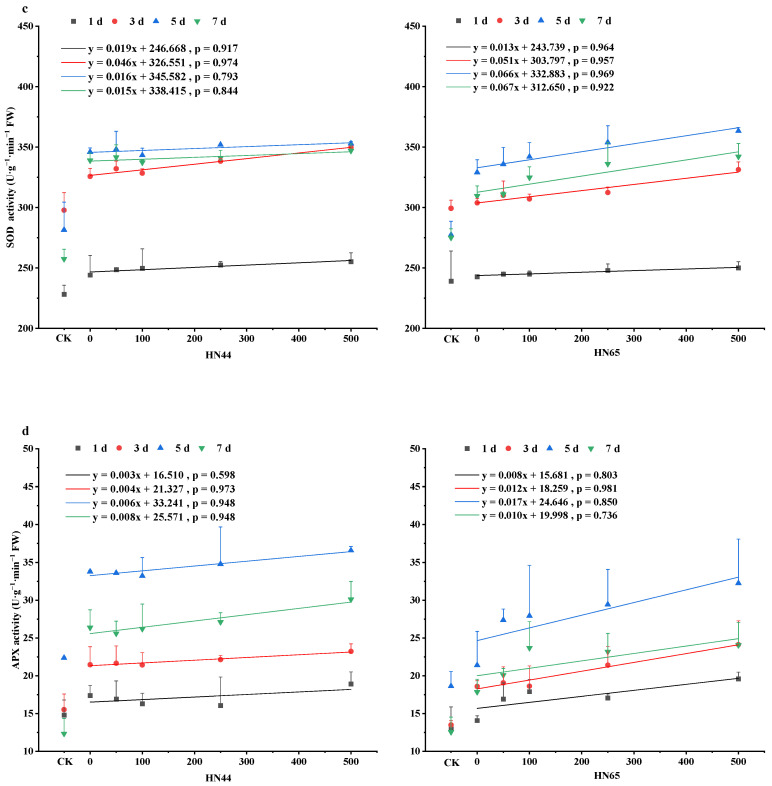
Changes in Antioxidant Enzyme activities during NO relieving drought stress. (**a**) peroxidase (POD) activity, (**b**) catalase (CAT) activity, (**c**) superoxide dismutase (SOD) activity, (**d**) ascorbate oxidase (APX) activity. The *X*-axis, CK represents the control, and the remaining numbers are SNP concentrations sprayed. The values (means ± SE) are the averages of three independent experiments.

As shown in Figure 3a, Drought stress increased peroxidase (POD) activity in HN44 by 6.32%, 39.10%, 43.14%, and 45.97% on days 1, 3, 5, and 7; POD activity in HN65 increased by −7.23%, 3.26%, 14.76%, and 14.76%. POD activity in HN44 showed an upward trend on day 1. On days 3, 5, and 7, S50 and S100 inhibited POD activity, and S250 and S500 increased POD activity. Compared with that for S0, POD activity increased by 14.25%, 19.05%, 21.28%, 38.29% in the S50, S100, S250, and S500 treatment groups on day 1. For the sensitive variety HN65, spraying SNP at 1, 5, and 7 days increased the activity of POD in soybean leaves during drought stress. At 3 days, POD activity decreased under S50 and S100 and reached the highest levels at 7 days (i.e., compared to that of S0, increases of 1.61%, 3.38%, 6.19%, 21.18% were observed in each treatment group). In HN44, POD activity was always greater than that of HN65. There was a significant difference in POD activity between the drought treatment and treatment with a high concentration of SNP for both varieties and low concentrations for HN44.

As shown in Figure 3b, Drought stress increased catalase (CAT) activity in HN44 by 11.21%, 63.32%, 71.47%, and 105.85% on days 1, 3, 5, and 7; CAT activity in HN65 increased by 35.99%, 19.30%, 54.60%, and 58.31%. CAT activity in HN44 leaves was reduced by S100 and S250 treatments on days 1 and 7 and by S50 on day 3. Compared with that in S0, CAT activity increased by 3.40%, 10.54%, 15.94%, and 28.10% in the S50, S100, S250, and S500 groups, respectively, on day 5. The activity of CAT in HN65 fell as the SNP content increased on day 1 and the S100 treatment reduced CAT activity on day 7. CAT activity increased with the increase of SNP concentration at 3, 5, 7 days. Compared with that of S0, the CAT activities in the S50, S100, S250, and S500 groups increased by 12.80%, 24.88%, 37.75%, and 53.69%, respectively.

As shown in Figure 3c, Drought stress increased superoxide dismutase (SOD) activity in HN44 by 6.98%, 9.39%, 22.89%, and 31.65% on days 1, 3, 5, and 7; SOD activity in HN65 increased by 1.52%, 1.52%, 18.64%, and 12.54%. SOD activity in leaves was basically stable in HN44 and was slightly lower under the S100 treatment than in other treatment groups at 3, 5, and 7 days. SOD activity at day 5 differed by 0.63%, −0.77%, 1.73%, and 2.02%, respectively, in the S50, S100, S250, and S500 groups compared with that in S0. SOD activity in HN65 generally showed an upward trend as the SNP content increased. Compared with that in S0, SOD activity in the other treatment groups increased by 2.05%, 3.92%, 7.53%, and 10.41%, respectively, on day 5. SOD activity in HN44 was greater than that of HN65 overall. The control group subjected to drought treatment at 5 and 7 days in HN65 showed significant differences in SOD activity from that of plants sprayed with different concentrations of SNP solutions.

As shown in Figure 3d, Drought stress increased ascorbate oxidase (APX) activity in HN44 by 17.38%, 38.24%, 51.01%, and 113.77% on days 1, 3, 5, and 7; APX activity in HN65 increased by 8.42%, 37.75%, 14.89%, and 42.31%.APX activity reached a peak on day 5. In HN44, the S50 and S100 treatments reduced APX activity in soybean leaves, and S250 reduced APX activity on day 1. On day 5, the activity of APX increased by −0.49%, −1.65%, 2.99%, and 8.33% for S50, S100, S250, and S500, respectively, compared with that in S0 and tended to decrease initially and increase thereafter. In the sensitive variety HN65, APX activity increased as the sprayed SNP content increased. APX activity for the S250 treatment was lower than that of the S100 treatment at 1 and 7 days. Compared with S0, the activity of APX in each treatment increased by 27.92%, 30.56%, 37.56% and 50.76% at day 5. The difference in APX activity between the drought treatment and HN65 sprayed with various concentrations of SNP was significant at 5 days and 7 days.

### 4.4. Changes in Osmotic Adjustment Ability in Soybean Leaves

**Figure 4:**
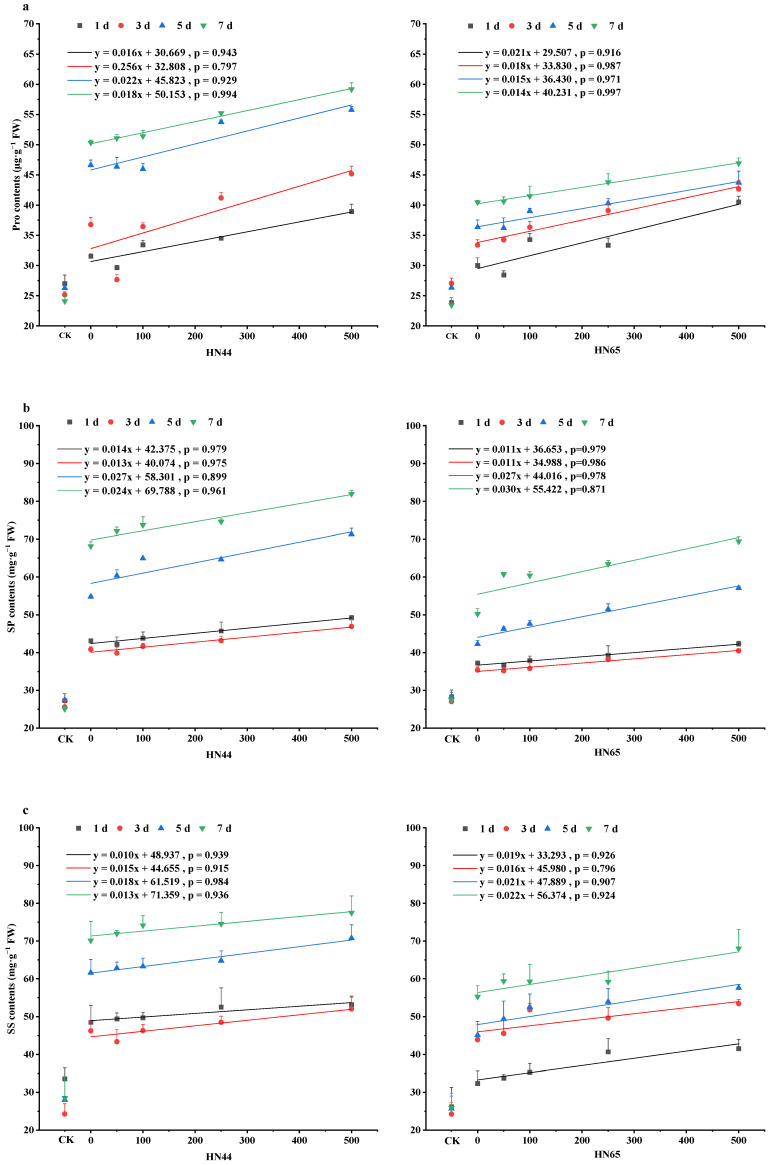
Changes in Pro, SP, and SS content during NO alleviation of drought stress. (**a**) proline (Pro) content, (**b**) soluble protein (SP) content, (**c**) soluble sugar (SS) content. The *X*-axis, CK represents the control, and the remaining numbers are SNP concentrations sprayed. Values (mean ± SE) are the averages of three independent experiments.

As shown in Figure 4a, the content of proline (Pro) tended decrease initially and then increase as the SNP content increased and the proline content increased gradually with the prolongation of time. Drought stress increased proline content in HN44 by 16.86%, 46.17%, 77.48%, and 108.96% on days 1, 3, 5, and 7; Proline content in HN65 increased by 26.03%, 23.39%, 38.00%, and 72.52%. The proline content of HN44 decreased under the S50 treatment on day 1, and the proline content decreased under the S100 and S250 treatments on days 3 and 5 and increased under other treatments. Compared with that of S0 on day 7, the Proline content in the medium increased the most, with estimates of 1.44%, 2.12%, 9.66%, and 17.50% for S50, S100, S250, and S500, respectively. The Proline content in HN65 decreased under the S50 treatment on days 1 and 5 and gradually increased in the other groups. Compared with S0, the Proline content in the S50, S100, S250, and S500 groups increased by 0.36%, 2.52%, 8.30%, and 16.00% in soybean leaves on day 7. The Proline contents in the two varieties tended to be stable on day 7, with greater Proline accumulation in HN44 than in HN65. There were significant differences, and there were significant differences in Proline accumulation between the S250 and S500 treatments compared with those in S0 on days 5 and 7.

As shown in Figure 4b, With the extension of time, the soluble protein contents tended to decrease initially and increase thereafter. The soluble protein contents generally increased with the increase in the SNP concentration. Drought stress increased soluble protein content in HN44 by 58.08%, 59.42%, 99.50%, and 172.20% on days 1, 3, 5, and 7; soluble protein content in HN65 increased by 8.42%, 37.75%, 14.89%, and 42.31%. The soluble protein content in HN44 was inhibited by S50 on days 1 and 3 and reached a peak on day 7. Compared with that in S0, the soluble protein contents increased by 5.80%, 8.13%, 9.43%, and 20.23% in the S50, S100, S250, and S500 groups, respectively. The soluble protein content in HN65 was inhibited on days 1, 3, and 7 by the S50 treatment, and the soluble protein content reached a peak on day 7. The soluble protein content in HN44 was greater than that in HN65.

As shown in Figure 4c, the content of soluble sugar (SS) in HN44 decreased first and then increased with the prolongation of time, and the content of soluble sugar in HN65 gradually increased with the prolongation of time. Drought stress increased soluble sugar content in HN44 by 58.08%, 59.42%, 99.50%, and 172.20% on days 1, 3, 5, and 7; soluble sugar content in HN65 increased by 31.46%, 30.98%, 49.16%, and 83.19%. The HN44 was in a suppressed state 3 days after treatment with S100, and the soluble sugar content in leaves was highest on day 7. Compared with S0, the soluble sugar contents in S50, S100, S250, and S500 increased by 2.56%, 5.69%, 6.26%, and 10.43%, respectively. The soluble sugar content of the sensitive variety HN65 was highest in leaves on day 7. Compared with that in S0, the soluble sugar content increased by 7.44%, 7.27%, 7.11%, and 23.14% in S50, S100, S250, and S500, respectively, and the differences among groups were significant. The concentration of soluble sugar in HN44 was generally greater than that in HN65.

### 4.5. Changes in MDA Contents under Different SNP Treatments

Drought stress significantly increased the malondialdehyde (MDA) content in plant leaves, and the MDA content increased with the prolongation of treatment time. Under drought stress, the MDA content in HN44 increased by 11.20%, 16.76%, 15.65%, and 43.88% on days 1, 3, 5, and 7 As the SNP concentration increased, the MDA contents of the HN44 gradually decreased. For the S500 treatment, MDA contents in HN44 decreased by 7.73%, 15.63%, 16.41%, and 13.80% compared with that in S0 on days 1, 3, 5, and 7, respectively.

**Figure 5:**
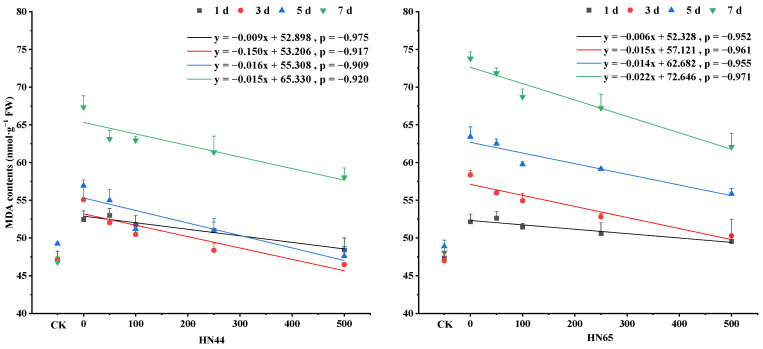
Changes in MDA content during NO alleviation of salt stress. MDA stands for Malondialdehyde. The *X*-axis, CK represents the control, and the remaining numbers are SNP concentrations sprayed. The values (means ± SE) are the averages of three independent experiments.

Under drought stress, the MDA content in HN65 increased by 10.13%, 24.22%, 29.70%, and 53.47% on days 1, 3, 5, and 7. As the SNP concentration increased, the MDA contents of the HN65 gradually decreased. For the S500 treatment, MDA contents in HN65 decreased by 4.97%, 13.83%, 11.92%, and 15.86% compared with that in S0 on days 1, 3, 5, and 7. These results indicated that NO reduces the MDA content in leaves, and the MDA content of the drought-resistant variety HN44 was lower than that of the sensitive variety HN65. (Figure 5).

### 4.6. Principal Component Analysis(PCA) and Multifactor Analysis of Variance

**Figure 6:**
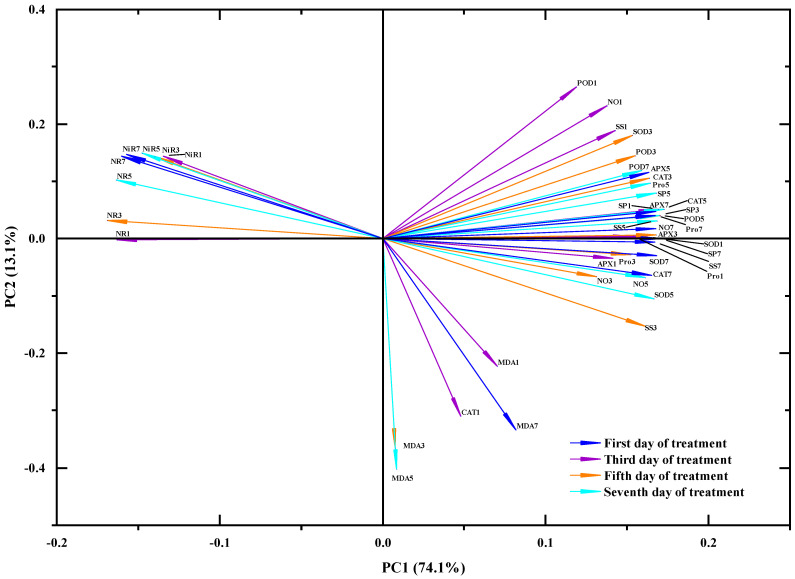
Principal Component Analysis. The projection of the line represented by a certain variable on PC1 and PC2 is the contribution of the variable to the sample separation. The longer the line, the larger the projection and the more significant the impact. The blue line represents the treatment on the first day, the purple line represents the treatment on the third day, the orange line represents the treatment on the fifth day, and the cyan line represents the treatment on the seventh day.

To better display the results of this experiment, principal component analysis was used to analyze the indicators of different days and the different treatments methods of the two varieties. The results are shown in Figure 6, PC1 accounts for 74.1% of the total variance of the model, and PC2 accounts for 13.1% of the total variance of the model, and the overall performance is good. It shows that there is a strong correlation between each index under the spraying of different concentrations of SNP, but the correlation between different soybean varieties is weak under the spraying of different concentrations of SNP. It indicated that the ability of the sensitive type HN65 to cope with drought stress increased after spraying SNP, which was similar to the drought resistance ability of the drought-resistant type HN44.

The independent effects of different treatments and days on the determination indexes were analyzed by Multifactor Analysis of Variance. It can be seen from Table 2 that the sig values of F values corresponding to different treatments were less than 0.05, indicating that different treatments had significant effects on the measured indexes. Similarly, the sig value of the F statistic of days was less than 0.05, indicating that the days also had a significant impact on the measured indicators. different treatments anddays represents the interaction between these two factors. The sig values of the F statistics corresponding to NiR and POD in HN44 and NR, APX, CAT, and POD in HN65 were all greater than 0.05, indicating that the interaction of different treatments and days had no significant effect on the determination index, but had a significant effect on the other indicators.

## 5. Discussion

This study aimed to evaluate the potential of SNPs in alleviating the inhibition of soybean physiology by drought stress. This paper shows that SNPs can alleviate soybean damage under drought stress conditions. After spraying SNP, the NO content in leaves increased significantly, consistent with previous results showing that spraying SNP on maize under drought stress can increase the NO content [31]. In this study, the nitrite reductase (NiR) and nitrate reductase (NR) activities in soybean leaves decreased in response to SNPs under the influence of drought. Kato et al. subjected lotus root nodules to salt stress and found that nitrogenase activity was inhibited by NO, and it was considered that the decrease in nitrogenase activity might be related to nitrate in root nodules, consistent with the results of this study [32]. This indicates that NO did not attenuate the negative effects of drought on NiR and NR. It is possible that during drought stress, NO is converted into nitrate and then the enzymatic activity is reduced to inhibit the reduction of nitrate [33], which is consistent with the results of this experiment. To sum up, spraying SNP increased the content of NO in plants and decreased the activities of NiR and NR in soybean. In soybean varieties with different sensitivity to drought, the activities of NiR and NR decreased more in drought-resistant varieties, which was related to the high content of NO in drought-resistant varieties.

According to previous studies, a large amount of ROS will be produced in soybeans under drought stress, which is adverse to soybean growth [34,35]. At this time, antioxidant enzymes are actively expressed in plants. Superoxide dismutase (SOD) in antioxidant enzymes can convert active oxygen species into hydrogen peroxide, and then peroxidase (POD), catalase (CAT), and ascorbate oxidase (APX) in antioxidant enzymes further convert hydrogen peroxide into substances with lower plant toxicity. In this study, drought treatment increased the activity of antioxidant enzymes. With the increase in NO content, the activity of antioxidant enzymes in soybean leaves showed an upward trend. Liu et al. [36] Refrigerated peaches and sprayed deionized water with NO. The results showed that NO enhanced the transcription levels of related genes of antioxidant enzymes, increased the activity of antioxidant enzymes, and prolonged the storage life. Khademi et al. [37] irrigated NO donors to garlic under salt stress. The results showed that salt stress increased the activities of SOD, POD, APX, and other antioxidant enzymes in garlic irrigated with SNP, which enhanced the viability of garlic under salt stress, which was consistent with the results of this experiment. The activity levels of APX and SOD were consistent with the trend in NO, with the highest peak at 5 days, clearly indicating that NO can regulate the activity of APX and SOD in a concentration-dependent manner. The activity levels of POD in HN65 in the CK, S0, and S50 groups were lower than that of normally watered plants on day 1, which may be due to the closure of stomata and gas exchange with the external environment after a pause in drought stress [38]. POD activity is substantially reduced by oxygen, and a high concentration of NO promotes the opening of stomata [39]. In response to NO, CAT activity showed a gradual increase over time [40]. Ji et al. [41] Sprayed NO donors on the leaves of cotton to increase the biosynthesis of the CAT gene in cotton under normal growth conditions and thereby increase CAT activity, consistent with the results of this experiment. To sum up, spraying SNP increased the activity of antioxidant enzymes in soybean and enhanced the ability of soybean to cope with drought stress. The activity of antioxidant enzymes changed greatly in soybean varieties with different sensitivity to drought.

Under drought stress, the malondialdehyde (MDA) content significantly increased [27], and after spraying SNP it remained at a low level of lipid peroxidation, alleviating oxidative damage to cell membranes. Hogg et al. [42] showed that NO effectively reduces membrane lipid peroxidation. When Sundararajan et al. [43] carried out drought stress on seedlings of Solanum lycopersicum, it was found that the intervention of NO reduced the content of MDA in plants and improved the ability of plants to cope with drought stress, which was consistent with the results of this experiment. To sum up, spraying SNP treatment can reduce the MDA content in the soybean body and reduce the peroxidation degree of soybean membrane lipid, and the drought-resistant soybean varieties with different degrees of drought sensitivity can reduce the MDA content more.

The proline (Pro) content in soybean leaves tended to decrease and then increase as the NO content increased and short-term drought and spraying low concentrations of SNP can reduce the accumulation of proline in plants. Jelonek et al. [44] sprayed a low concentration of SNP on cucumber and observed a reduction in proline, consistent with the results of this study. Prochazkova et al. [15]. were subjected to heat stress and sprayed with NO scavengers. The results showed that the content of NO and proline decreased in faba beans receiving NO scavengers, indicating that NO can be used as a signal molecule to induce proline expression. Wei et al. [45] studied the relationship between NO and soluble protein in tobacco. A low concentration of NO promoted the accumulation of soluble protein, and a high concentration of NO inhibited the accumulation of soluble protein. These results were contrary to the results of this experiment, and the difference can probably be attributed to differences between species and sampling times. Hao et al. [46] sprayed exogenous SNP on dry Ginkgo leaves and showed that soluble sugar (SS) in the leaves increased at 250 μmol/L, similar to the results of this experiment. The soluble sugar content was related to the MDA content. Nemati et al. [47] showed that fructan reduces the MDA content in wheat (Triticum aestivum). Under drought stress, fructan will break down into soluble sugar, which may explain why soluble sugar is inhibited at low NO contents, while high contents of NO promote ROS scavenging and increases soluble sugar. In summary, spraying SNP treatment increased the content of osmotic adjustment substances in soybeans and reduced the water potential difference in soybeans. The soybean varieties with different degrees of sensitivity to drought generally had more osmotic adjustment substances in sensitive varieties.

In this study, nitroprusside was used as the NO slow-release agent to stabilize the NO level in soybean leaves, and a control group with normal watering was included for comparison. However, key hormones, e.g., example abscisic acid, were not evaluated. Subsequent experiments should focus on the determination of hormones and other indicators of the plant response to stress.

## 6. Conclusions

Damage to soybean leaves caused by drought stress was reduced by the accumulation of NO, osmolytes, and increases in antioxidant activity. The ability to withstand drought was consistently better in HN44 than in HN65. This finding indicated that NO could enhance the drought resistance of soybean, and spraying SNP at 500 μmol/L had the best effect on alleviating drought in the flowering stage. Therefore, when precipitation is insufficient in actual production, SNP can be sprayed to enhance the drought resistance of soybean.

## Figures and Tables

**Table 1:** Test treatment method. PEG-6000 was dissolved in water and then watered in pots, and SNP was sprayed on soybean leaves by front and back spray. CK—control; S0—drought + 0 μmol/L SNP; S50—drought + 50 μmol/L SNP; S100—drought + 100 μmol/L SNP; S250—drought + 250 μmol/L SNP; S500—drought + 500 μmol/L SNP.

	Constituents	PEG-6000	SNP (μmol/L)
Treatment			
CK		0	0
S0		15%	0
S50		15%	50
S100		15%	100
S250		15%	250
S500		15%	500

**Table 2:** Multifactor Analysis of Variance. Analysis of variance, using the F test method, the F value in the result represents a specific value obtained by the F test formula, and the corresponding *p* value is obtained according to the numerical table, which is sig. The sig value < 0.05 indicates that it affects the result, otherwise it has no effect. Different treatments are CK—control; S0—drought + 0 μmol/L SNP; S50—drought + 50 μmol/L SNP; S100—drought + 100 μmol/L SNP; S250 –drought + 250 μmol/L SNP; S500—drought + 500 μmol/L SNP. Days are 1 d, 3 d, 5 d, 7 d. Index are nitric oxide (NO), nitrite reductase (NiR), nitrate reductase (NR), peroxidase (POD), catalase (CAT), superoxide dismutase (SOD), ascorbate oxidase (APX), proline (Pro), soluble protein (SP), soluble sugar (SS), malondialdehyde (MDA).

		Different Treatments		Days		Different Treatments and Days	
Variety	Index	F	Sig	F	Sig	F	Sig
HN44	NO	173.001	<0.01	396.733	<0.01	18.203	<0.01
	NiR	51.955	<0.01	33.215	<0.01	0.768	0.705
	NR	166.089	<0.01	24.92	<0.01	3.143	0.001
	POD	33.903	<0.01	44.348	<0.01	1.162	0.333
	CAT	18.854	<0.01	63.081	<0.01	1.995	0.036
	SOD	45.224	<0.01	256.572	<0.01	3.313	0.001
	APX	25.104	<0.01	116.587	<0.01	2.415	0.011
	Pro	757.019	<0.01	1001.968	<0.01	58.858	<0.01
	SP	750.399	<0.01	1102.429	<0.01	50.863	<0.01
	SS	130.832	<0.01	129.369	<0.01	5.773	<0.01
	MDA	82.765	<0.01	188.203	<0.01	10.8	<0.01
HN65	NO	127.699	<0.01	297.073	<0.01	10.057	<0.01
	NiR	39.499	<0.01	154.797	<0.01	55.242	<0.01
	NR	56.685	<0.01	19.408	<0.01	1.245	0.273
	POD	8.804	<0.01	27.147	<0.01	1.041	0.433
	CAT	7.782	<0.01	16.526	<0.01	0.942	0.527
	SOD	22.682	<0.01	183.575	<0.01	3.462	0.001
	APX	12.8	<0.01	23.472	<0.01	0.768	0.704
	Pro	257.096	<0.01	107.024	<0.01	7.367	<0.01
	SP	750.399	<0.01	1102.429	<0.01	50.863	<0.01
	SS	130.832	<0.01	129.369	<0.01	5.773	<0.01
	MDA	194.069	<0.01	459.815	<0.01	18.867	<0.01

## Data Availability

Not applicable.
